# A Sparse Neural Code for Some Speech Sounds but Not for Others

**DOI:** 10.1371/journal.pone.0040953

**Published:** 2012-07-16

**Authors:** Mathias Scharinger, Alexandra Bendixen, Nelson J. Trujillo-Barreto, Jonas Obleser

**Affiliations:** 1 Max Planck Research Group “Auditory Cognition”, Max Planck Institute for Human Cognitive and Brain Sciences, Leipzig, Germany; 2 Institute of Psychology, University of Leipzig, Leipzig, Germany; 3 Cuban Neuroscience Center, Havana, Cuba; University of British Columbia, Canada

## Abstract

The precise neural mechanisms underlying speech sound representations are still a matter of debate. Proponents of ‘sparse representations’ assume that on the level of speech sounds, only contrastive or otherwise not predictable information is stored in long-term memory. Here, in a passive oddball paradigm, we challenge the neural foundations of such a ‘sparse’ representation; we use words that differ only in their penultimate consonant (“coronal” [t] vs. “dorsal” [k] place of articulation) and for example distinguish between the German nouns *Latz* ([lats]; bib) and *Lachs* ([laks]; salmon). Changes from standard [t] to deviant [k] and vice versa elicited a discernible Mismatch Negativity (MMN) response. Crucially, however, the MMN for the deviant [lats] was stronger than the MMN for the deviant [laks]. Source localization showed this difference to be due to enhanced brain activity in right superior temporal cortex. These findings reflect a difference in phonological ‘sparsity’: Coronal [t] segments, but not dorsal [k] segments, are based on more sparse representations and elicit less specific neural predictions; sensory deviations from this prediction are more readily ‘tolerated’ and accordingly trigger weaker MMNs. The results foster the neurocomputational reality of ‘representationally sparse’ models of speech perception that are compatible with more general predictive mechanisms in auditory perception.

## Introduction

Recent decades of research in auditory neuroscience have led to a better understanding of cortical mechanisms subserving the perception of single speech sounds, of combinations of speech sounds, and of whole words (e.g. [1]). However, the precise nature of speech sound representations (pertaining to their neural code) is still a matter of debate, although there is consensus that these neural codes be flexible enough to guarantee successful recognition in a variety of different, and sometimes difficult, listening situations (e.g. [2], [3], [4]).

Amongst various linguistically-based approaches, some consider cognitive speech sound representations to be entirely faithful to the physical signal and therefore stipulate very detailed and fine-grained units (e.g. Exemplars, [5], [6], [7], [8], [9]), sometimes linked to episodic memory [10]. If such models held, flexibility of speech sound representations would have to be achieved through learning of many exemplars in many different situations. In sharp contrast, proponents of *sparse representations* assume that on the level of speech sounds, only contrastive or otherwise not predictable information is stored in long-term memory (e.g. [11], [12], [13]), adhering to the linguistic concept of “underspecification” (reviewed in [14]). A key assumption is that speech sounds are built of abstract features that they do not directly correspond to their acoustic properties. Together with the assumption that some of these features lack a precise neural specification, this approach is an attractive one for a neural architecture of speech: It ensure flexibility of neural speech codes by means of sparseness and readily accounts for so-called assimilation asymmetries between different places of articulation. For instance, “coronal” sounds such as [n] very often assimilate to the place of articulation of their following neighbors (e.g. lean bacon > lea*m* bacon due to labial [b]) and are therefore assumed to not carry any place of articulation information in their neural code. In contrast, non-coronal sounds such as [m] hardly ever assimilate to the place of articulation of their neighbors (e.g. rum toffee > ru*n* toffee due to coronal [t] is hardly ever encountered). As a result, these sounds are considered to be based on a more precise, rigid neural code.

Here, we set out to test the assumption that due to the more sparse representation of coronals, their predictive value in auditory speech perception is smaller than for non-coronals (dorsals). An example for a dorsal consonant can be found in the initial position of ‘call’, i.e. [k], while a coronal consonant in initial position would be [t] in ‘tall’. We consider the predictive value as the inference that is being made on the basis of a particular speech sound feature, such as coronality. We further assume that this information is used for predicting upcoming speech sounds even when these are unattended, such as in a passive oddball paradigm. It has previously been shown that contingent feature relations between sound sequences are rapidly extracted [15], [16] in such paradigms, and that these extracted regularities are used to predict future sound events [17], [18], [19], [20], [21]. We expect that from a coronal sound, the system cannot derive a precise prediction with regard to coronality or non-coronality because of the sparse coronal representation, while a specified dorsal sound provides a strong prediction for non-coronality. A viable way of measuring the strength of a prediction is to measure the size of the prediction error signal generated when the prediction is violated [18]. Following this logic, we would expect a deviation from a coronal sound to elicit weaker change detection responses than a deviation from a dorsal sound.

A neurophysiological marker that lends itself readily to the assessment of such changes is the Mismatch Negativity (MMN), an automatic and pre-attentive brain response to auditory regularity violations [22], [23], [24]. It is elicited in passive oddball designs where the evoked response to a rare deviant sound usually differs from that to a frequent standard sound. MMN research on speech sounds [25], syllables [26], [27], [28], [29] and words [30], [31], [32] has provided evidence that the magnitude of the MMN is not solely dependent on the acoustic standard/deviant difference (for a direct comparison, see [33], [34], [35]). Experiments with speech stimuli have shown that the MMN is modulated by the familiarity of the listener with the respective stimulus or stimulus sequence [30], [36], [37], [38], [39], [40], [41], by its context [42], [43], or lexicality (i.e. whether it is a word or a pseudo-word, cf. [30], [31], [32], [44], [45]) and by its frequency of occurrence [46].

Proponents of underspecification [47], [48], [49] particularly focus on sound-specific characteristics and provided neurophysiological evidence for sparse neural codes of vowels. For any parsimonious explanation, however, it is important that these findings would generalize to all speech sounds, i.e. to vowels *and* consonants. Further, the particular model by [11] assumes that long-term memory representations are independent of the sound’s position in a word. For these reasons, we extend the existing research by looking at *word-final* coronal and non-coronal (dorsal) *consonants* in the German words *[lats]* (bib) and *[laks]* (salmon) with a comparable number of semantic meanings and controlled other linguistic characteristics (frequency and familiarity measures are provided in Table 1).

**Table 1:** Phone, diphone, and word frequencies of the stimulus set.

	coronal [t]	dorsal [k]
phoneme C	–3.84	–3.40
diphone Cs	–3.81	–3.23
diphone aC	–3.37	–3.30
word	–1.10	–1.10

All values are reported as log-values of occurrences in parts-per-million, according to CELEX ([95]). For phoneme and diphone frequencies, the values are normalized with respect to the cumulative frequency of all German lemmas containing these sequences. The respective consonant is underlined.

**Figure 1:**
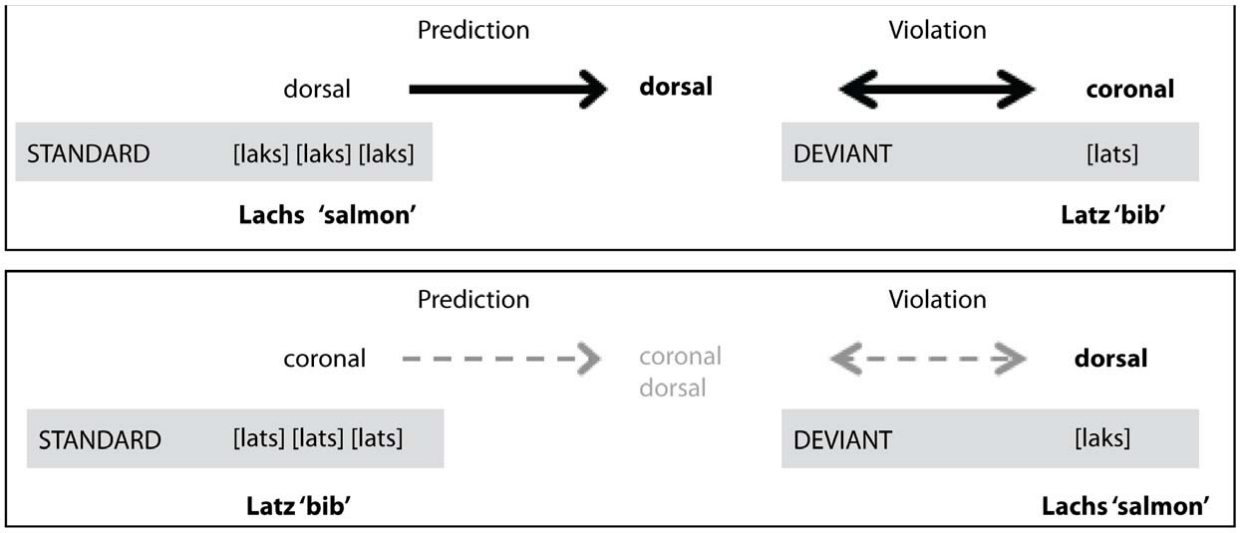
Top: Inferences generated by the dorsal standard *[laks]* (salmon) and violation through the coronal deviant *[lats*] (bib). Due to the high inference value of the dorsal standard, the violation is predicted to be severe. Bottom: Inferences from the coronal standard *[lats]* are weak, and thus, the violation through the dorsal deviant *[laks]* is predicted to be less severe.

Note that overall, frequency and familiarity measures differ only slightly between the two nouns, *[laks]* and *[lats]*. Importantly, these differences should be negligible in comparison to [41], which leads us to assume – on the basis of previous evidence for the extraction of feature-based regularities from standard sound sequences [16], [18], [19], [20], [21], [50] – that the abstract property pertaining to place of articulation, rather than word or consonant frequency, is used for predicting subsequent sound events in a passive oddball paradigm. Further, we speculate that the predictive values extracted from the coronal and dorsal standards differ, such that the prediction violation through the respective deviants is asymmetric. A more detailed sound (such as a dorsal) bears a high inference value with respect to the featural dimension “place of articulation”. The prediction that the next sound in a standard sequence is similarly specified should be high, and the violation of this prediction is severe. In contrast, a more fuzzy representation of a coronal sound has a low inference value, and the prediction regarding a particular place of articulation in a subsequent standard is low. Consequently, a violation of this weaker prediction is less severe. This is illustrated in Figure 1. We thus predict that in a passive oddball sequence of standard *[laks]* – deviant *[lats]* (dorsal – coronal), the resulting MMN will be larger than in the sequence of standard *[lats]* – deviant *[laks]* (coronal – dorsal).

## Materials and Methods

### Participants

Twenty right-handed native speakers of German with normal hearing (50% females, mean age 25, SD 2.6) took part in the study. They were drawn from the subject pool of the Max Planck Institute for Human Cognitive and Brain Sciences, Leipzig, and received monetary compensation for their participation. All participants gave written informed consent before they participated in this study, which was approved by the Max Planck Institute for Human Cognitive and Brain Sciences, Leipzig. One participant had to be excluded due to a hearing ailment (tinnitus), one participant due to technical problems (EEG data of one block were not recorded), and one additional participant due to low signal-to-noise ratio in the EEG (only 56% usable data). This resulted in a total of seventeen participants (41% females, mean age 25.4, SD 2.2) whose data were used for the event-related potential (ERP) analyses.

### Stimuli

**Figure 2:**
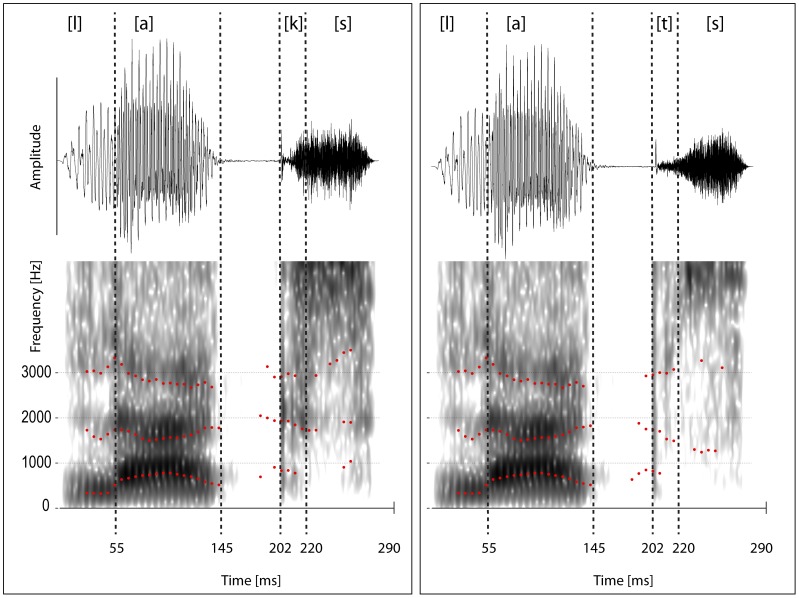
Wave forms and spectrograms with formant tracking of the two words Lachs *([laks],* ‘salmon’, left panel) and Latz *([lats],* ‘bib’, right panel). Note that the crucial difference between the consonants [k] and [t] is seen in the second resonance frequency (F2). Reported formant values were averaged across durations starting from the pre-release phase (at 188 ms) and ending at the boundary to [s] (at 220 ms).

The German nouns *Lachs* ([laks]; salmon) and *Latz* ([lats]; bib) were recorded from a professional native speaker in several repetitions, using a Røde NT 55 microphone (amplitude resolution 16 bits, sampling rate 44.1 kHz). The speaker maintained a consistent neutral intonation. The most consistent pronunciations with approximately the same durations for the initial consonant-vowel sequence [la] were selected for further processing, and cross-spliced in the following way. The final consonant clusters [ts] and [ks] were cut off the initial [la] sequences, using the sound application PRAAT [51]. The two remaining [la] sequences were trimmed to the same duration of 202 ms. Subsequently the [la] sequences were morphed together (point-by-point averaging) in order to avoid cues of co-articulation prior to the release of the word-final consonant. Finally, the final clusters [ts] and [ks] were added to the morphed and co-articulation-free [la] sequence. This provided us with two acoustic stimuli that were identical up to the release of the consonant [t] (*[lats]*) and [k] (*[laks]*), i.e. during the first 202 ms. The words with a total duration of 290 ms mainly differed in the 2^nd^ formant of the transition from the closure into the consonant release (see Table 2 and Figure 2, cf. [52]).

**Table 2:** Acoustic properties of final consonant clusters.

	Intensity C [dB]	Intensity [s] [dB]	F2 transition [Hz]	F3 transition [Hz]
[laks]	68	72	1936	2909
[lats]	61	71	1731	2954

Intensity values are given in dB (∼dB SPL); the second and third formant values stem from the transition period of the vowel [a] into the release of the respective consonant (underlined).

Acoustic characteristics of the stimuli were determined in more detail by an intensity and formant analysis, carried out in the phonetic software tool PRAAT [51]. For the formant analysis, a fast-Fourier analysis was calculated, using a 25 ms Hanning window that was moved along the time dimensions in 5 ms steps. Formant values are reported for the transition between [a] and the following consonant, i.e. [k] or [t], respectively.

After cross-splicing, stimuli were calibrated to an average level of loudness equaling 60 dB SPL. This was done separately for [la] and the respective endings, [ts] and [ks], in order to guarantee the same loudness up to the point of deviation, i.e. after [la]. Onsets and offsets (10 ms) were multiplied with raised cosine ramps in order to eliminate acoustic artifacts.

### Design

The two words *[laks]* and *[lats]* were distributed over two conditions where they occurred either as standard (p = 0.875) or as deviant (p = 0.125), i.e. condition I consisted of standard *[laks]* and deviant *[lats]*, whereas condition II consisted of standard *[lats]* and deviant *[laks]*. In each condition, there were 960 stimuli, i.e. 840 standard and 120 deviant presentations. The sequence of standards and deviants was pseudo-randomized, such that there were at least 3 standards after each deviant. Stimuli were presented with a stimulus onset asynchrony (SOA) of 800 ms. This resulted in a total condition duration of 12.8 minutes.

Conditions were split up into two blocks of 6.4 minutes, with short breaks in-between. The order of conditions was randomized, while the four blocks reported here (two per condition) directly followed each other. Each block started with six additional standards that were not analyzed any further. The EEG recording session also included separate, additional blocks that were based on the same stimulus material, and either followed or preceded the blocks of this experiment (counter-balanced across participants). The additional blocks underwent an analysis with a different focus and are reported elsewhere. As they were also recorded in a passive setting, and included overall equal proportions of *[laks]* and *[lats]* stimuli (excluding differential refractoriness of the two stimuli), the additional blocks are unlikely to have influenced the electrophysiological recordings reported here. Altogether, the entire session (including breaks, preparation time, and a post-test identification task) lasted for about 3 hours.

### Experimental Procedure

During the EEG experiment, participants were seated in an acoustically shielded room, approximately 60 cm in front of a screen that was used for presenting a silent movie during the passive listening, a strategy commonly applied during MMN experiments [53].

Auditory stimuli were presented over Sony MDR XD100 headphones at a comfortable listening level (∼60 dB SPL), using the stimulus presentation software PRESENTATION (Neurobehavioral Systems Inc). Participants’ electrophysiological responses were recorded from 64 Ag-AgCl-electrodes (58 scalp electrodes, 2 mastoids, 2 electrodes for horizontal and 2 for vertical electrooculograms) on a Brain Vision EEG system. Scalp electrodes were arranged according to the 10% extension of the international 10–20 system [54], [55]. The nose was used as online reference. Brain electric responses were recorded with a sampling rate of 500 Hz. Electrode impedances were kept below 5 kΩ. The recording pass-band ranged from DC to 250 Hz.

### Post-EEG Identification Task

After EEG recording, participants completed a two-alternative forced-choice (2-AFC) identification task in which they had to identify the two stimuli *[laks]* and *[lats]*. This was done in order to ensure that the experimental stimuli were in fact distinguishable. In this experiment, participants listened to a random sequence of *[laks]* and *[lats]* stimuli with an SOA of 800 ms. The experiment finished after both *[laks]* and *[lats]* had been presented at least 50 times. Participants had to identify each word by a corresponding button press. The assignment of button responses to either *[laks]* or *[lats]* was counter-balanced across participants; half of them pressed X for *[laks]* and Z for *[lats]*, the other half pressed X for *[lats]* and Z for *[laks]*. The experiment was again delivered using the PRESENTATION software and lasted for about 5 minutes. Performance was analyzed in terms of response times and identification accuracy separately for *[lats]* and *[laks]* stimuli and compared between the stimulus types by means of paired-sample, two-tailed Student’s t tests.

### Electroencephalographic Data Analysis

Electroencephalographic data were processed with EEGlab [56] in a MatLab environment (MathWorks GmbH). Data were filtered offline with a band pass filter of 0.5–30 Hz (1813 point Kaiser windowed sine FIR filter, Kaiser beta = 5.65). Data recorded at the eye channels were bipolarized offline to yield vertical and horizontal electroocular activity (EOG), respectively. For averaging, epochs of 400 ms were extracted from the continuous EEG recording, including a 100-ms pre-deviation baseline. Note that the deviation occurred 202 ms after stimulus onset (i.e., at the release of the final consonant, see Figure 2), thus the epochs lasted from 102 to 502 ms relative to stimulus onset. Epochs with an amplitude change exceeding 100 µV on any channel were rejected from further analysis; on average 90.2% of the trials could be retained. In addition to the block-initial six standards, each standard immediately following a deviant was excluded from further analyses because such standards are known to elicit a small mismatch response compared to the rest of the standards [57], [58].

Epochs for the four stimulus types (*[lats]* standard, *[laks]* standard, *[lats]* deviant, *[laks]* deviant) were averaged separately to form ERPs. ERP difference waves were derived from physically identical stimuli (*[lats]* deviant minus *[lats]* standard, *[laks]* deviant minus *[laks]* standard) in order to remove stimulus-specific ERP effects [59]. The MMN component was quantified as the average ERP amplitude from 135 to 215 ms following deviation onset. Measurements were taken from the Fz electrode. MMN amplitudes for the two conditions (*[lats]* difference, *[laks]* difference) were separately tested against zero using one-sample, two-tailed Student’s t tests. MMN amplitudes were then compared between conditions using a paired-sample, two-tailed Student’s t test.

For studying the scalp topographies in the MMN latency range, ERP voltage distributions were transformed into scalp current density (SCD) distributions. The SCD analysis provides a reference-independent measure of the scalp distribution that sharpens the voltage distribution as volume-conducted signals from distant regions of the head are attenuated [60]. Therefore, the SCD distribution is primarily determined by electrical activity within a short distance of each electrode (i.e., superficial cortical tissue; [60]), allowing to assess local contributions to the observed ERP response. The present SCD analyses followed the spherical spline surface Laplacian algorithm of [61]. The radial current at a given location on the surface (Laplacian) was computed as the second spatial derivative of the interpolated voltage distribution [61]. The maximum degree of the Legendre polynomials was chosen to be 50, and the order of splines (m) was set to 4. A smoothing parameter of 10^−5^ was applied.

Aiming to reveal the cortical generators of the MMN, brain electrical tomography analyses were applied by means of variable resolution electromagnetic tomography (VARETA; [62], [63]). With this technique, sources are reconstructed by finding a discrete spline-interpolated solution to the EEG inverse problem: estimating the spatially smoothest intracranial primary current density (PCD) distribution compatible with the observed scalp voltage distribution. This allows for point-to-point variation in the amount of spatial smoothness and restricts the allowable solutions to the gray matter, based on the probabilistic brain tissue maps available from the Montreal Neurological Institute [64]. The procedure minimizes the possibility of „ghost sources“, which are often present in linear inverse solutions [65]. A 3D grid of 3244 points (voxels, 7 mm grid spacing), representing possible sources of the scalp potential, and the recording array of 60 electrodes (excluding the 4 eye channels) were registered with the average probabilistic brain atlas developed at the Montreal Neurological Institute. Subsequently, the scalp potentials in the MMN latency range were transformed into source space (at the predefined 3D grid locations) using VARETA. Statistical parametric maps (SPMs) of the PCD estimates were constructed based on a voxel by voxel Hotelling T^2^ test against zero (group statistics; based on N = 17) in order to localize the sources of the component separately for the two conditions. For all SPMs, Random Field Theory [66] was used to correct activation threshold for spatial dependencies between voxels. Results are shown as 3D activation images constructed on the basis of the average brain.

### Behavioral Follow-up Experiment

In order to examine the behavioral relevance of the electrophysiological differences in deviance detection obtained in the main experiment, an additional behavioral experiment was conducted. Participants (N = 20, 56% females, mean age 26.6, SD 3.7) were recruited from the subject pool of the Max Planck Institute for Human Cognitive and Brain Sciences, Leipzig. They were paid for their participation and had not previously participated in the EEG study.

The behavioral test used the same design as the passive-listening paradigm of the MMN study, except that fewer trials were used and participants had to press a button whenever they perceived a deviant. In the first condition, participants were presented with 400 standards (*[laks]*) and 50 deviants (*[lats]*), while the second condition consisted of the same number of *[lats]* standards and *[laks]* deviants. Before each condition, participants completed a practice run with 32 trials in order to familiarize with the task. They were required to press the left mouse button as fast and accurately as possible whenever the sequence of standards (minimally three in a row) was interrupted by the corresponding deviant. As before, stimuli were delivered over headphones at a comfortable listening level (∼60 dB SPL), using the stimulus presentation software PRESENTATION (Neurobehavioral Systems Inc.). The experiment lasted for about 20 minutes.

Data of one participant had to be excluded from the analysis because he misunderstood the instruction and thus erroneously responded after every stimulus. Detection performance was analyzed by means of signal detection theory [67], [68]. All responses within 100 to 1200 ms after a deviant stimulus were considered hits. With an SOA of 800 ms, this implies that target responses could still be made while the next stimulus had already been presented. This requires a slightly modified version of calculating the sensitivity index d′. We adopted the procedure described by Bendixen and Anderson [69], evaluating false alarms relative to the number of time intervals without targets that are equally long as the accepted response intervals (see also [70]). Prior to d′ calculation, hit and false alarm rates were adjusted to 1–1/(2N) when they were actually 1 and to 1/(2N) when they were actually 0, with N being the number of observation periods [68], [71], [72]. Sensitivity indices d′ as well as mean response times were compared between the detection tasks by means of paired, two-tailed Student’s t-tests.

For both behavioral tests, response times were measured relative to the onset of the consonant [k] or [t], respectively. All significant t-test results are reported with Cohen’s *d″* for dependent samples.

## Results

### Behavioral Performance: Identification

All participants were able to identify the *[lats]* and *[laks]* stimuli. The percentage of identification errors did not significantly differ between *[lats]* (mean 5.31%, standard error of mean [s.e.m.] 4.30%) and *[laks]* (mean 6.26%, s.e.m. 10.17%, t(16) = −0.387, p = 0.704). Likewise, response times did not significantly differ between *[lats]* (mean 399.72 ms, s.e.m. 71.84 ms) and *[laks]* (mean 388.24 ms, s.e.m. 53.59 ms), *t*(16) = −0.977, *p* = 0.343). Note that the variance in error rates was higher for *[laks]* than for *[lats]*. This is attributable to one participant who often misinterpreted *[laks]* as *[lats].* Although the explanation for this response pattern is unclear, it is important to note that the non-significant differences between error rates and response times for *[laks]* and *[lats]* were replicated when excluding this participant from the analysis.

### Analysis of the Mismatch Negativity (MMN)

**Figure 3:**
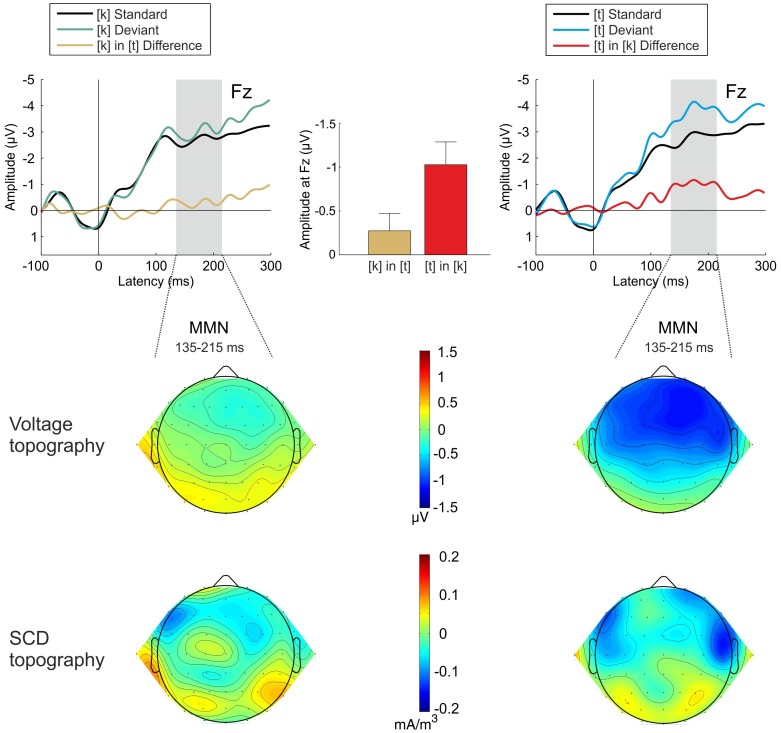
Top row: Grand-average ERPs elicited by standard (black line) and deviant (blue line) *[laks]* (left panel) and *[lats]* (right panel) stimuli. The deviant-minus-standard difference waveforms are given in red. The latency range for measuring the MMN component (135–215 ms) is marked in gray. Mean MMN amplitudes for the two contrasts are given in the top middle panel, error bars indicate standard errors of mean. Middle row: Voltage topographies in the MMN latency range. Bottom row: SCD topographies in the MMN latency range. Smoothing parameter was 10^−7^ for the voltage distribution and 10^−5^ for the SCD distribution.

Deviant *[lats]* stimuli elicited a clear MMN component (Figure 3, right panel) whose presence was statistically confirmed at Fz [*t*(16) = –3.969, *p*<0.01, *d″* = −0.963]. The activity in the MMN latency range at Fz was not significant for deviant *[laks]* stimuli (Figure 3, left panel) [*t*(16) = –1.370, *p* = 0.190]. It was, however, significant when re-referencing against the average of the mastoids as recommended by [73] [*t*(16) = –2.390, *p*<0.05, *d″* = −0.580].

Notably, the MMN amplitude at Fz was significantly stronger for the *[lats]* than for the *[laks]* difference waveform [*t*(16) = -2.197, *p*<0.05, *d″* = -0.533]. The MMN voltage topography displayed in Figure 3 shows that this amplitude difference is present at all frontocentral channels. The SCD topography suggests that this may be due to the enhanced activation of a right-hemispheric generator. This assumption was verified in a next step of the analysis by means of source localization with the VARETA approach.

### Source Localization of the Mismatch Negativity (MMN)

**Figure 4:**
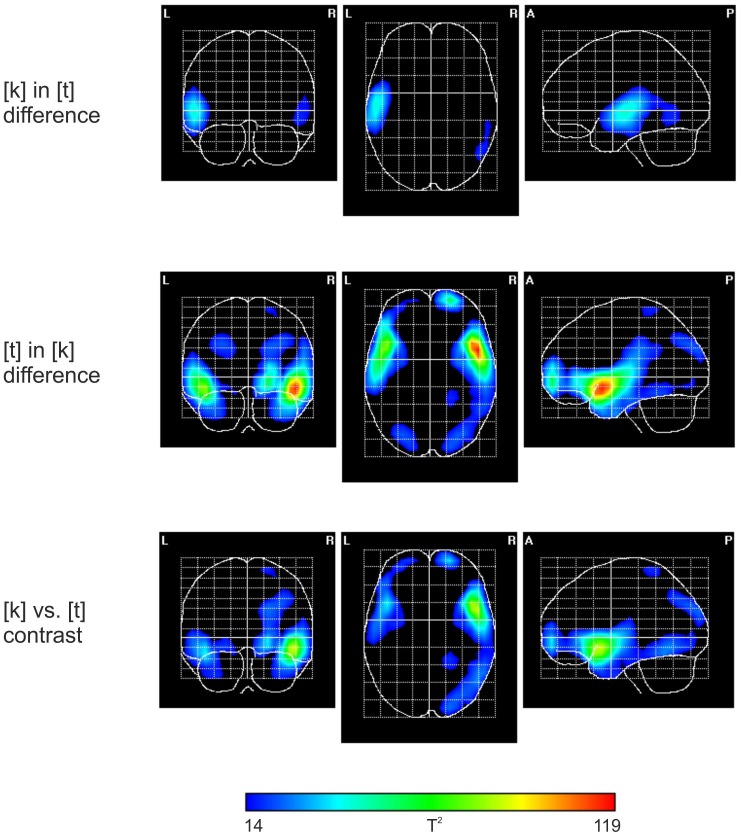
VARETA source localizations of the MMN responses to *[laks]* (top) and *[lats]* (middle), and significant activation differences between the *[laks]* and *[lats]* solutions (bottom). Significant centers of activation are color-coded with warmer colors for higher probability values (one-way ANOVA; thresholded to p<0.0001).

The statistical results of the source-space reconstruction for the activity in the MMN latency range are displayed in Figure 4. Source space results are consistent with those of the sensor space (SCD) analysis in showing an enhancement in right superior temporal cortex activity for *[lats]* deviants as compared to *[laks]* deviants.

A direct statistical comparison (Figure 4 bottom) shows that the significant activation difference between the *[lats]* and *[laks]* scalp potentials in the MMN latency range is mainly due to this stronger right-hemispheric contribution.

### Behavioral Follow-up Results: Deviance Detection

The perceptual sensitivity to detect the deviant coronal *[lats]* within a dorsal *[laks]* context was significantly higher (d′ = 4.5) than vice versa (d′ = 3.2; *t*(18) = 5.26, *p*<0.001, *d″* = –1.2063). This asymmetry was also visible in the reaction times. The reaction time of detecting the deviant *[lats]* was significantly shorter (371 ms) than the reaction time of detecting the deviant *[laks]* (451 ms, *t*(18) = –4.21, *p*<0.001, *d″* = 0.965). Note that the relatively short reaction times can be attributed to the speed of presentation, using an SOA of 800 ms. Thus, the deviant *[lats]* was detected more accurately and faster than the deviant *[laks]*.

### General Discussion

In this MMN study, we examined differences in the neural signatures of the German consonants [k] and [t] to test the neural reality of a ‘sparse’ representational code. First, we found an asymmetry in the MMN responses to the minimal word pair *[laks]* vs. *[lats]* that contain these consonants in a word-final cluster. In particular, the MMN elicited by the deviant *[lats]* with coronal [t] was of greater magnitude than the MMN elicited by the deviant *[laks]* with dorsal [k].

Second, the source localization of the MMN response between approximately 130 and 200 ms after deviation onset showed an enhanced right-temporal contribution for *[lats]* as compared to *[laks]*. Third, the electrophysiological pattern was paralleled by behavioral measures from the deviance detection experiment. Here, the detection of the deviant *[lats]* was more accurate and faster than the detection of *[laks]*.

The MMN pattern in our experiment supports an approach that attributes [t] and [k] in *[lats]* and *[laks]* different predictive values arising from differences in their neural codes. The results cannot be explained on the basis of acoustic properties or phonotactic probabilities of the stimulus material, as will be discussed in detail below.

### Differences in Neural Codes

We interpret the asymmetric MMN pattern as evidence for different long-term memory representations of the consonants [t] vs. [k] that distinguish between the two nouns *[lats]* and *[laks]*. In particular, we claim that the neural code of coronal [t] is less specific and hence provides a less restricted perceptual prediction based on the recurring standard *[lats],* with regard to subsequent stimulus presentation in an oddball design. As a result, the sudden occurrence of a [k] sound (in *[laks]*) provides a relatively weak prediction violation. In contrast, dorsal [k] is more specific with regard to place of articulation, which – so we argue – requires a precise neural code that additionally involves spectrally fine-tuned areas of the right-hemispheric auditory cortex (e.g. [74]), as discussed in the next section in greater detail. The specificity of [k] therefore generates a narrow and strong prediction for subsequent stimuli, such that the violation by the deviant *[lats]* elicits an enhanced MMN response.

Importantly, the prediction differences between *[laks]* and *[lats]* were also reflected in the behavioral deviance detection task. Here, we found that participants were faster and more accurate to detect *[lats]* in the context of *[laks]* than vice versa. The accuracy and reaction time advantage of *[lats]* depended on the expectation for *[laks]* generated in that particular condition. Crucially, if *[lats]* occurred with the same probability as *[laks],* as was true in the identification task, no behavioral asymmetry was observed. This supports our claim that the asymmetry depends on the difference in prediction, being stronger for [k] in *[laks]* than for [t] in *[lats]*. It is also possible that participants in the identification task used an A-not-A-strategy, i.e. responded on the basis of a [t]-non-[t] or a [k]-non-[k] contrast, and therefore showed no response time or error rate differences between the two words. We argue that if participants followed this response strategy, it was caused by the lack of predictability in the identification task, where *[laks]* and *[lats]* occurred with the same probability. In contrast, the repetition of standards in the deviance detection task resulted in specific predictabilities, which were not only beneficial but indispensable for task performance.

Finally, one can observe a pattern of faster responses to *[lats]* in the deviance detection than in the identification task, whereas responses to *[laks]* were slower in the deviance detection than in the identification task. We claim that this asymmetry reflects the contribution of prediction error detection to task performance. For a strong prediction violation (from *[laks]* to *[lats]*), prediction error detection is so efficient as to even speed up task performance relative to an identification task with no predictive context, whereas for a weak prediction violation (from *[lats]* to *[laks]*), prediction error detection is less efficient and thus prolongs task performance.

### From Representation to Prediction

Our assumption that differences in speech sound representations lead to different predictions extracted from standard sequences and are violated by the respective deviants [16], [18], [19], [20], [21], [50] is also compatible with the predictive coding account of MMN generation [17], [24], [75], [76], [77], [78]. It embraces the finding that human brain function is characterized by predictive processing [76], [79]. The existing context of sensory input shapes anticipatory responses to future events. Predictive processing is considered crucial for auditory cognition in general, and for speech perception in particular [80].

Regarding the MMN, the predictive coding approach is based on the model adjustment hypothesis [21], [81], [82] according to which the MMN results from the need to update an acoustic model of the environment in order to incorporate (or assimilate) the respective deviant. The model is instantiated by a sequence of standard sounds and generates inferences regarding future sound events, that is, a continuation of the standard sequence. Importantly, the model can have high or low inference values, which means that a deviant is less readily or more readily incorporated in the model [21]. Put differently, the model can be more or less confident in inferring future sound events, and consequently will show larger mismatch responses if a highly confident inference is violated. Within the predictive coding framework, the repetition of standards results in a continuous reduction of prediction errors, and we suggest that predictions emerging from more specific speech sound representations are stronger than predictions emerging from sparsely represented speech sounds. Thus, dorsal [k] in standard position sets up strong predictions regarding the place of articulation feature of upcoming sounds and at the same time, by not violating these predictions, the prediction error is continuously reduced. Upon encountering the coronal deviant [t], however, the model fails to suppress the prediction error, and an MMN response is generated. This response is stronger than in the reverse case, in which the coronal [t] sets up the predictions in standard position. Due to its less specific representation, its inference value regarding the place of articulation of upcoming sounds is lower, and the prediction error reduction is less efficient. As a result, the dorsal deviant [k] provides a weaker violation of prediction, or, in different terms, a less severe failure to suppress the prediction error.

Although our experiment suggests different neural codes for [t] and [k], our data cannot provide a definite response of whether these codes, pertaining to speech categories, exert top-down influences on lower auditory areas, or whether there are already categorical and abstract representations in these auditory areas. There is recent neuroimaging work providing evidence for perceptual and categorical representations in the vicinity of Heschl’s gyrus and the Planum temporale (e.g. [83]). The present MMN sources in left and particularly, right-temporal areas are compatible with these findings. It therefore seems reasonable to assume that the asymmetric MMN pattern in fact directly reflects representational differences in speech sounds, i.e. differences in their neural codes.

### Hemispheric Biases in Deviant Detection

The stronger MMN response to the deviant *[lats]* was accompanied by larger activity in right-temporal areas that could be detected on the basis of a VARETA source analysis between 135 and 215 ms post deviance onset (Figure 4). With all due caution concerning the accuracy of EEG source localization, we consider this as enhanced activation of a right-temporal source. We interpret this enhanced activation on the background of the model adjustment hypothesis [21], [81], [82]. As discussed before, this hypothesis takes the MMN to reflect the need of updating an acoustic model of the environment in order to incorporate (or assimilate) a particular deviant. The ‘effort’ for this incorporation should depend on how strongly the respective standard predicts a particular feature to occur. Here, dorsal *[laks]* sets up a strong prediction with regard to place of articulation, mediated by acoustic-phonetic differences in F2. In order to incorporate the deviant *[lats]* with slight differences in F2 in its consonant [t], we hypothesize that a more detailed model update is necessary, compared to the reverse situation where *[lats]* sets up a weak prediction, and the incorporation of the dorsal deviant *[laks]* necessitates a less detailed model update. Note that this hypothesis considers the model update to reflect an acoustically mediated place of articulation conflict resolution, that is, the underlying symmetric acoustic contrast is modulated by inferences derived from the specificity of place of articulation information. We further argue that a more detailed model update benefits from additional neural resources that are dedicated to process spectral details.

Previous neuroimaging and electrophysiological studies suggest that right-temporal networks fulfill this task. Regions in the right superior temporal sulcus and gyrus are particularly responsive to spectral processing (e.g. [74], [84], [85], [86]). Some studies even suggest that the MMN has stronger sources in the right than in the left hemisphere (e.g. [87], [88]).

Taken together, the enhanced right-hemispheric source in our experiment may be related to higher demands of spectral processing in the deviant *[lats]* condition for the integration of the deviant in the acoustic model of the standard [21], [81].

Note that the right-hemispheric source might also reflect an entirely non-phonological processing component. This should not be problematic for our interpretation - as shown elsewhere, the MMN reflects both *phonological* and *auditory* processes (e.g. [35]). An enhancement in auditory processing however actually supports our claim that lexical information is less informative in coronal [t]. Note also that the enhanced right-temporal source is not at odds with the expectation that place of articulation information is processed in the left rather than the right hemisphere. In fact, we observed left-hemispheric sources in both deviant conditions. We claim that the enhanced right-hemispheric source does not reflect place of articulation processing per se (or differences thereof), but the resolution of an acoustic-to-phonology mapping conflict. The original model anticipates that such conflict resolution can actually occur on an acoustic-phonetic level [11].

### Alternative Explanations

While our experiments reported here provide positive evidence for differences in specificity between coronal [t] and dorsal [k], some researchers found MMN patterns that do not appear to support the unspecific status of coronal speech sounds and interpret their findings in alternative ways (e.g. [89], [90], [91]). The above-mentioned studies report enhanced MMN amplitudes to labial (i.e. non-coronal, e.g. [b]) as compared to coronal deviants (e.g. [d]), contrary to the expectation that coronal deviants should elicit stronger responses. It is important to note, though, that in these cases, the stimulus material did not consistently involve lexical material of the participants’ mother tongue. Scharinger and colleagues [89] used vowel-consonant-vowel sequences that did not constitute existing words, while Rivera-Gaxiola and colleagues [90], [91] presented Hindi sounds to English listeners. It seems likely that the MMN response in these cases was more strongly affected by the familiarity of the listeners with the respective words or sounds than by the sound-based properties alone. Alternatively, one could argue that the contradicting results, involving stronger effects for labials than for coronals in both cases, has to do with a peculiarity of labials, viz. their greater visual salience. That visual salience may override phonetically-based asymmetries appears to be reflected in a set of eye tracking experiments [92]. Here, auditory instructions with target words starting with coronal [t] or labial [p] served as triggers to click on one of four visual probes that either did or did not have a relation to the auditory target. The rationale was that words with labial onsets ought to activate words with non-labial onsets, but words with coronal onsets should not activate words with labial onsets. As a result, the amount of looks on non-labial words should have been higher than the amount of looks on labial words. The results – measured in amount of looks – only showed trends in this direction. However, this is not surprising since the visual selection always offered an optimal candidate, sometimes even co-indexed by a visual symbol. Thus, it is not clear to what degree this experiment showed a modality-independent activation of speech units by auditory inputs.

There are two important alternative explanations (one of them acoustic, the other frequency-related) that need to be considered for the present set of data. We do, however, argue that both of them are implausible, as illustrated below.

#### Acoustic effects

On the most basic level, the MMN asymmetry in this experiment could be solely determined by the acoustics of the stimuli. While this alternative explanation is unlikely since we based the calculation of the MMN on the same acoustic tokens (identity MMN, [59]) and thereby mitigated acoustic concerns, it might still be possible that the asymmetry reflected an asymmetry in formant changes between standard and deviant. On average, [k] has a higher F2 value than [t] in the relevant time window, such that the change from *[laks]* to *[lats]* is accompanied by a decrement of resonance frequency, while the change from *[lats]* to *[laks]* is accompanied by an increment of resonance frequency. Only few studies explicitly examined the direction of changes in the frequency domain [93], [94]. Crucially, Peter and colleagues [94] found that when the deviant had a higher frequency than the standard, the resulting MMN response was larger than in the reverse case, i.e. when the deviant had a lower frequency than the standard. This suggests that increments in frequency between standards and deviants should cause enhanced MMN responses, a finding that is opposite to what we observed in our data. In contrast to that study, we found that an increment in F2 frequency between standard and deviant (*[lats]* – *[laks]*) was accompanied by an attenuated MMN. Hence, an acoustic explanation of the asymmetry in our experiment is not feasible.

#### Frequency of occurrence effects

Regarding a possible confound of word occurrence frequency, previous cross-linguistic research on phoneme and word representations has provided evidence for a familiarity effect in MMN designs [36], [37], [38], [39]. In general, MMN responses to phonemes or words familiar to the listener (due to learning or the occurrence in the corresponding native language) are stronger than responses to unfamiliar speech sounds or words. Recently, this familiarity effect has been shown to apply within a language, in that words with a higher frequency of occurrence elicited larger MMN effects [46]. A frequency-based explanation does not hold for our data, though. Note that the two stimuli, *[laks]* and *[lats]*, had the same frequency of occurrence (as determined from CELEX, [95]). Therefore, our asymmetry cannot be explained by the frequency of occurrence of our stimuli.

Finally, Bonte and colleagues [41] showed that the MMN is sensitive to statistical regularities in speech sound sequences. They provided evidence that in Dutch, a consonant sequence with a high phonotactic probability elicited a stronger MMN than a consonant sequence with a low phonotactic probability. Phonotactic probability was assessed by the log-value of the number of words containing the particular consonant sequence in relation to their summed frequencies of occurrences. Thus, perhaps, the asymmetry in our experiment was caused by differences in phonotactic probability of these sequences. When applied to our data, the sequence with the coronal consonant had in fact slightly higher frequency values (corresponding to phonotactic probability, see Table 1) than the sequence with the dorsal consonant. However, care must be taken in comparing phonotactic probability of Bonte’s study with phonotactic probability in the present study. First, the crucial difference between *[lats]* and *[laks]* in our experiment is the consonant following the vowel [a], such that most informative phonotactic probability should be carried by the sequences [at] and [ak]. These diphone frequencies hardly differed at all. Second, the differences were much more pronounced in Bonte’s experiment than in our experiment. Third, and most importantly, Bonte [41] examined phoneme sequences in pseudowords, and across possible syllable boundaries, while we investigated monosyllabic real words. It is not obvious how phonotactic probability within real words would affect the MMN response in passive oddball designs. This question must certainly await future research. At the moment, however, a phonotactic probability account of our data is not feasible.

We are further aware that it is difficult to derive generalizations from a single contrast, involving two different words only. While this is a technical constraint of MMN studies, necessitating a large number of repetitions in a reasonable amount of time, our results can be taken as converging evidence for less specific representations of coronals, if compared to behavioral and neurophysiological studies that showed the same findings in other sounds, words, or languages [11], [12], [13], [47], [48], [49], [96], [97], [98]. We therefore conclude that the predictive coding approach together with the assumption of sparse neural codes provides the best account of our data.

### Conclusions

We have provided evidence for differences in specificity in the neural codes subserving the representation of the speech sounds [t] and [k]. We found larger MMN amplitudes and enhanced right-hemispheric activity in response to the deviant *[lats]* (with [t]) preceded by the standard *[laks]* (with [k]) than in the reverse case. The most parsimonious explanation for this asymmetry is a difference in the predictive value carried by the standards, with [k] generating a more specific and thus more concrete neural prediction than [t] regarding the next speech sound in the oddball sequence. The notion of differences in representational specificity of speech sounds therefore appears as a fruitful starting point for further investigations of how this specificity affects predictive processing in auditory perception.
